# Clinical Roles of Lung Volumes Detected by Body Plethysmography and Helium Dilution in Asthmatic Patients: A Correlation and Diagnosis Analysis

**DOI:** 10.1038/srep40870

**Published:** 2017-01-18

**Authors:** Jian Luo, Dan Liu, Guo Chen, Binmiao Liang, Chuntao Liu

**Affiliations:** 1 Department of Respiratory Diseases, West China School of Medicine and West China Hospital, Sichuan University, Chengdu, China; 2 Department of Critical Care Medicine, West China School of Medicine and West China Hospital, Sichuan University, Chengdu, China; 3 Department of Gerontology, Sichuan Academy of Medical Sciences and Sichuan Provincial People’s Hospital, Chengdu, China

## Abstract

Roles of lung volumes in asthma remain controversial. We aimed to evaluate the efficacy of lung volumes in differentiating asthma severity levels. Consecutive outpatients with chronic persistent asthma were enrolled, and body plethysmography (BP) and helium dilution (HD) were performed simultaneously to extract RV%pred, TLC%pred, and RV/TLC. Significant negative correlations were found between FEV_1_%pred and RV%pred (r = −0.557, P < 0.001), TLC%pred (r = −0.387, P < 0.001), and RV/TLC (r = −0.485, P < 0.001) measured by BP, as well as difference in volumes between these two techniques (ΔRV%pred, ΔTLC%pred and ΔRV/TLC). In mild and moderate asthma, AUC of RV%pred detected by BP and ΔTLC%pred was 0.723 (95%CI 0.571–0.874, P = 0.005) and 0.739 (95%CI 0.607–0.872, P = 0.002) with sensitivity and specificity being 79.41% and 88.24%, and 65.22% and 56.52% at cut-off of 145.40% and 14.23%, respectively. In moderate and severe asthma, AUC of RV%pred detected by BP and ΔTLC%pred was 0.782 (95%CI 0.671–0.893, P < 0.001) and 0.788 (95%CI 0.681–0.894, P < 0.002) with sensitivity and specificity being 77.78% and 97.22%, and 73.53% and 52.94% at cut-off of 179.85% and 20.22%, respectively. In conclusion, lung volumes are reliable complement of FEV_1_ in identifying asthma severity levels.

Asthma is a common, chronic airway disease with an increasing prevalence ranging from 1.8% to 14.5% of the population as varied by country and population^1^,^2^. It has been widely acknowledged that airway inflammation plays a central role in the development of asthma, which is clinically characterized by a pattern of respiratory symptoms and variable expiratory airflow limitation^3^. In spite of the extensive investment in treatment, much of the underlying pathogenesis of asthma remains unknown, and preventable deaths caused by asthma continue to occur, especially in patients with recurrent severe exacerbations^4^. Therefore, early and accurate identification of patients with risk of exacerbation and efficacious application of treatment may improve survival and quality of life in asthmatics.

Variable airflow limitation confirmed by positive bronchodilator reversibility test or positive bronchial challenge test is one of the prerequisite in establishing a diagnosis of asthma and in identifying potential exacerbation, of which forced expiratory volume in one second (FEV_1_) is the most commonly used and studied^5^,^6^. However, FEV_1_ is not necessarily associated with severity^7^. In recent decades, lung volumetric parameters such as residual volume (RV) and total lung capacity (TLC) have been demonstrated to be potential measures in evaluating asthma severity levels and treatment responses^8^,^9^,^10^,^11^. In addition, lung volumes especially RV reported in most studies were measured by body plethysmography (BP). BP remains as the gold standard but may lead to an overestimate of RV when in the presence of severe obstruction, due to the potential for the gas within all regions of the lung and airways to undergo unequal and asynchronous compression or decompression during panting maneuvers^12^,^13^,^14^. Helium dilution (HD) is an alternative method for measuring alveolar volume but may lead to an underestimate because gas contained within the poorly ventilated regions is not included in the helium estimate of lung volume^13^,^14^,^15^. In a small study by Woolcock and colleagues, functional residual capacity (FRC) and TLC were found to be significantly higher by plethysmography than those by dilution method, and the differences between these methods were the greatest when the FEV_1_ was lowest, and these differences decreased during clinical recovery^16^. Therefore, we hypothesized that the difference in volume between these two methods may provide additional clinical value in identifying individuals with differing asthma severity.

To test this hypothesis, we conducted a prospective correlation and diagnosis analysis in an attempt to further assess the correlation between lung volumes and FEV_1_, and the values of individual lung volumes as well as the corresponding differences between the two methods in distinguishing asthma severity.

## Results

### Demographics

A total of 93 patients (48 male and 45 female) were enrolled in our final analysis, of which 23 (24.73%) had mild asthma, 34 (36.6%) had moderate asthma, and 36 (38.7%) had severe asthma. The mean age of patients with mild, moderate, and severe asthma was 52.0, 53.4, and 54.4 years old, respectively; but there was no significant difference (*P* = 0.777). No difference was observed in the duration of asthma (4.5 ± 4.0 vs. 4.7 ± 2.5 vs. 6.1 ± 2.9 years, *P* = 0.083) or smoking history (6.7 ± 8.7 vs. 10.5 ± 12.4 vs. 10.9 ± 10.8 pack*year, *P* = 0.321) between groups (Table 1). Despite a significant difference of gender between different asthmatic severity groups (6 vs. 18 vs. 24, *P* = 0.010), the between group difference was only significant between patients with mild and severe asthma (*P* = 0.003) (Table 1).

### Lung volumes differences between BP and HD and among different asthmatic severity levels

**Figure 1:**
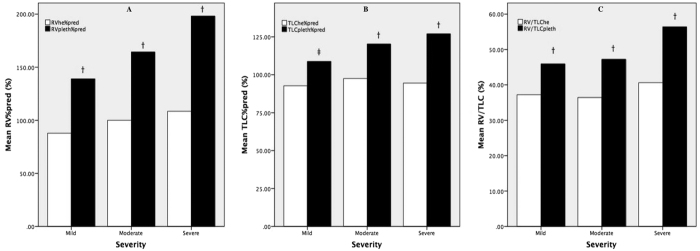
Comparison of RV%pred, TLC%pred and RV/TLC between BP and HD in different asthmatic severity levels. Comparison of RV%pred (**A**), TLC%pred (**B**), and RV/TLC (**C**) measured by BP (black) and HD (white) showed that RV%pred, TLC%pred, and RV/TLC were significantly higher in BP than in HD in all asthmatic severity levels. BP, body plethysmography; HD, helium dilution method; RV%pred, predicted percentage of residual volume; RV/TLC, ratio of residual volume to total lung capacity; TLC%pred, predicted percentage of total lung capacity. ^†^P < 0.001; ^‡^P = 0.001.

Predicted percentage of RV (RV%pred) measured by BP was significantly higher than that by HD regardless of asthma severity (mild: 139.0 ± 38.1% vs. 87.8 ± 18.6%, *P* < 0.001; moderate: 164.2 ± 24.5% vs. 100.0 ± 20.0%, *P* < 0.001; severe: 198.1 ± 37.8% vs. 108.5 ± 28.6%, *P* < 0.001) (Fig. 1A). A similar pattern was seen in predicted percentage of TLC (TLC%pred) (mild: 108.9 ± 15.6% vs. 92.7 ± 13.8%, *P* = 0.001; moderate: 120.2 ± 12.2% vs. 97.5 ± 11.3%, *P* < 0.001; severe: 127.0 ± 13.3% vs. 94.5 ± 13.5%, *P* < 0.001) (Fig. 1B) and RV/TLC (mild: 45.9 ± 7.5% vs. 37.2 ± 8.0%, *P* < 0.001; moderate: 47.2 ± 7.2% vs. 36.4 ± 7.8%, *P* < 0.001; severe: 56.4 ± 9.1% vs. 40.6 ± 8.4%, *P* < 0.001) (Fig. 1C).

We also found a significant increasing trend of RV%pred (139.0 ± 38.1% vs. 164.2 ± 24.5% vs. 198.1 ± 37.8%, *P* < 0.001), TLC%pred (108.8 ± 15.6% vs. 120.2 ± 12.2% vs. 127.0 ± 13.3%, *P* < 0.001), and RV/TLC (45.9 ± 7.5% vs. 47.2 ± 7.2% vs. 56.4 ± 9.1%, *P* < 0.001) measured by BP, rather than HD, as asthma severity levels (Table 1). In addition, a similar elevation of differences between BP and HD in predicted percentage of RV (ΔRV%pred), TLC (ΔTLC%pred) and RV/TLC (ΔRV/TLC) was also observed.

### Correlation between lung volumes and FEV_1_%pred

**Figure 2:**
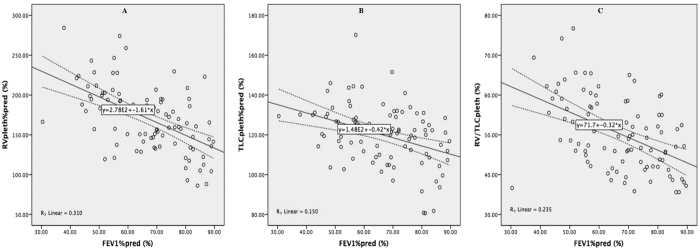
Scatter plots of RVpleth%pred, TLCpleth%pred and RV/TLCpleth according to FEV1%pred. Scatter plot of RVpleth%pred (**A**), TLCpleth%pred (**B**), and RV/TLCpleth (**C**) and FEV_1_%pred showed that RVpleth%pred, TLCpleth%pred, and RV/TLCpleth were negatively correlated with FEV_1_%pred with a *R*^*2*^ of 0.310, 0.150, and 0.235, respectively. FEV_1_%pred, predicted percentage of forced expiratory volume in one second; RVpleth%pred, predicted percentage of residual volume measured by body plethysmography; RV/TLCpleth, ratio of residual volume to total lung capacity measured by body plethysmography; TLCpleth%pred, predicted percentage of total lung capacity measured by body plethysmography.

**Figure 3:**
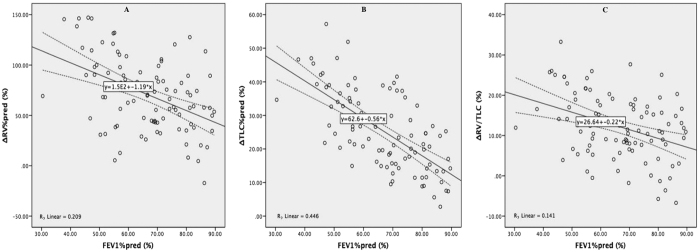
Scatter plots of ΔRV%pred, ΔTLC%pred and ΔRV/TLC according to FEV1%pred. Scatter plot of ΔRV%pred (**A**), ΔTLC%pred (**B**), and ΔRV/TLC (**C**) and FEV_1_%pred showed that ΔRV%pred, ΔTLC%pred, and ΔRV/TLC were negatively correlated with FEV_1_%pred with a *R*^*2*^ of 0.209, 0.446, and 0.141, respectively. FEV_1_%pred, predicted percentage of forced expiratory volume in one second; ΔRV%pred, predicted percentage of difference of residual volume between body plethysmography and helium dilution method; ΔRV/TLC, difference of ration of residual volume to total lung capacity between body plethysmography and helium dilution method; ΔTLC%pred, predicted percentage of difference of total lung capacity between body plethysmography and helium dilution method.

Significant negative correlations between FEV_1_%pred and RV%pred (*r* = −0.557, *P* < 0.001), TLC%pred (*r* = −0.387, *P* < 0.001), and RV/TLC (*r* = −0.485, *P* < 0.001) measured by BP, as well as ΔRV%pred (*r* = −0.457, *P* < 0.001), ΔTLC%pred (*r* = −0.668, *P* < 0.001), and ΔRV/TLC (*r* = −0.375, *P* = 0.002) were found (Table 2) and further illustrated in scatter plots (Figs 2 and 3). Nevertheless, such a correlation was not identified between FEV_1_%pred and TLC%pred (*r* = 0.089, *P* = 0.396) and RV/TLC (*r* = −0.176, *P* = 0.091) measured by HD, except for RV%pred (*r* = −0.245, *P* = 0.018).

### Lung volumes in distinguishing asthmatic severity levels

**Figure 4:**
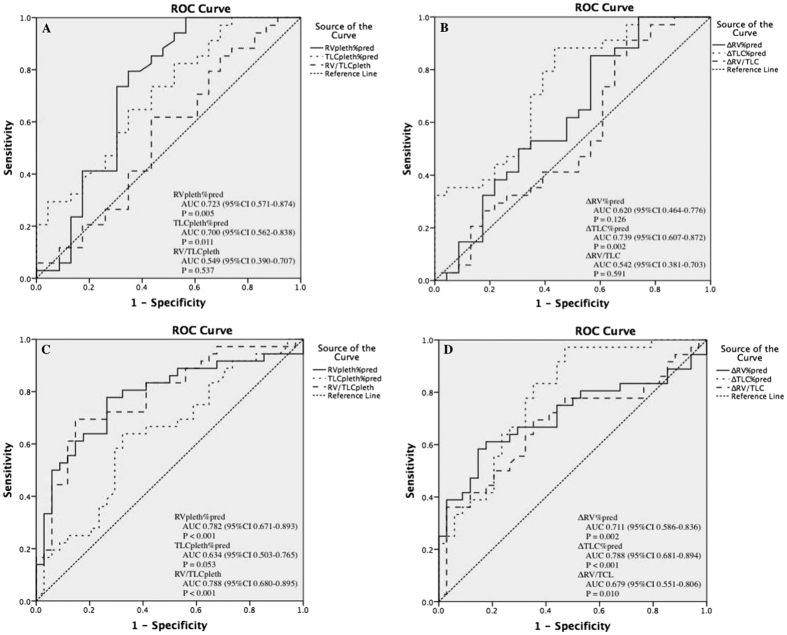
ROC curves of RVpleth%pred and ΔRV%pred, TLCpleth%pred and ΔTLC%pred, and RV/TLCpleth and ΔRV/TLC between mild and moderate, and moderate and severe asthma. ROC curves of RVpleth%pred (solid line), TLCpleth%pred (narrow dashed line) and RV/TLCpleth (wide dashed line) (**A** and **C**), as well asΔRV%pred (solid line), ΔTLC%pred (narrow dashed line) and ΔRV/TLC (wide dashed line) (**B** and **D**) in differentiating mild and moderate asthma, and moderate and severe asthma. AUC, area under the curve; CI, confidence interval; ROC curve, receiver operating characteristic curve; RVpleth%pred, predicted percentage of residual volume measured by body plethysmography; RV/TLCpleth, ratio of residual volume to total lung capacity measured by body plethysmography; TLCpleth%pred, predicted percentage of total lung capacity measured by body plethysmography; ΔRV%pred, predicted percentage of difference of residual volume between body plethysmography and helium dilution method; ΔRV/TLC, difference of ration of residual volume to total lung capacity between body plethysmography and helium dilution method; ΔTLC%pred, predicted percentage of difference of total lung capacity between body plethysmography and helium dilution method.

In distinguishing mild and moderate asthma, area under the curve (AUC) of RV%pred, TLC%pred and RV/TLC was 0.723 (95% confidence interval (CI) 0.571–0.874), 0.700 (95%CI 0.562–0.838) and 0.549 (95%CI 0.390–0.707), respectively; and significant differences were found in RV%pred (*P* = 0.005) and TLC%pred (*P* = 0.011) but not in RV/TLC (*P* = 0.537) (Fig. 4A). With regard to volumes, a significant difference was only found in ΔTLC%pred (AUC 0.739, 95%CI 0.607–0.872, *P* = 0.002) (Fig. 4B). Similarly, in discriminating moderate and severe asthma, we found significant differences in RV%pred (AUC 0.782, 95%CI 0.671–0.893, *P* < 0.001) and RV/TLC (AUC 0.788, 95%CI 0.680–0.895, *P* < 0.001) (Fig. 4C), as well as in ΔRV%pred, ΔTLC%pred, and ΔRV/TLC (Fig. 4D).

A cut-off of 145.4% and 179.9% in RV%pred exhibited a sensitivity and specificity of 79.41% and 77.78%, and 65.22% and 73.53%, respectively, in identifying different asthma severity levels; while a cut-off of 14.2% and 20.2% in ΔTLC%pred was estimated to have a sensitivity and specificity of 88.24% and 97.22%, and 56.52% and 52.94%, respectively, to retrieve moderate and severe asthma from mild and moderate asthma. (Table 3).

## Discussion

In our study, we found that lung volumes including RV%pred, TLC%pred and RV/TLC measured by BP were significantly higher than those measured by HD, and that the volumes measured by BP as well as the difference in volume between these techniques were positively correlated with increasing severity of asthma but negatively correlated with FEV_1_%pred. Furthermore, we also identified that RV%pred measured by BP and ΔTLC%pred could reliably distinguish both mild and moderate asthma and moderate and severe asthma with a high AUC and sensitivity.

BP measures thoracic gas volumes (TGV) on the basis of Boyle’s Law, which states that, under isothermal conditions, the product of gas volume and pressure is constant at any given moment, and results in an equation of TGV = −(ΔV/ΔP) × P_A2_ × (P_A1_/P_B_), of which ΔV is the change in volume of the thorax before and after compression or rarefraction of the gas in thorax, ΔP is the change in the alveolar pressure measured at the airway opening under conditions of no flow during the panting maneuver, P_A1_ and P_A2_ are the alveolar pressure before and after compression or rarefraction of the gas in thorax under the assumption that pressure measured at the airway opening is representative of alveolar pressure, and P_B_ indicates the barometric pressure^17^. In comparison, HD measures lung volume from communicating regions of the lung only, and the FRC at the time the subject is connected to the spirometry apparatus of a known volume (Vapp) and helium fraction (F_He1_) is calculated from the helium fraction at the time of equilibration (F_He2_) as the following equation: FRC = Vapp × (F_He1_ − F_He2_)/F_He2_^12^,^18^. Therefore, lung volumes measured by HD rely on the gas volume exhaled by the patient; and consequently gas contained within the poorly ventilated regions is not incorporated in the helium estimate of lung volume, leading to higher volumes measured by BP compared with HD in patients with gas trapping^14^,^17^,^19^. Previous studies have also demonstrated that lung volumes measured by BP may be more sensitive than HD in distinguishing different levels of current asthma status^16^,^20^. In our study, we also found such a difference in lung volumes between BP and HD, and the difference between the two methods further increased with increasing severity, which we speculate to be a result of an increasing gas trapping as demonstrated by the decreasing FEV_1_%pred in our study.

FEV_1_ is commonly used as a gold standard to diagnose chronic airway diseases, including asthma, and to evaluate their severity in accordance with the measurements of bronchodilator reversibility test or bronchial provocation test^3^,^21^,^22^. Nevertheless, these classifications were derived from expert opinion and were not validated in clinical studies. An increasing number of clinical observations and studies have demonstrated that FEV_1_ is poorly related to symptoms^23^. Bacharier and his colleagues found in a prospective cohort study on 219 asthmatic children that FEV_1_%pred was not significantly different between severity levels of asthma (99.6% vs. 97.2% vs. 101.0% vs. 93.7% in mild intermittent and persistent, and moderate and severe persistent asthma, respectively, P = 0.3)^7^. Mahut and his colleagues divided 180 asthmatic children with documented airflow reversibility into three groups of severe exacerbation, asthma symptoms without severe exacerbation, and asymptomatic asthma, and found that FEV_1_%pred tested before or after bronchodilator did not differ significantly between groups (pre-bronchodilator: 94 ± 15 vs. 96 ± 12 vs. 98 ± 15, P = 0.53; post-bronchodilator: 105 ± 13 vs. 108 ± 10 vs. 107 ± 12, P = 0.59)^24^. In addition, Stănescu and his colleagues found a lung functional pattern with decreased VC and FEV_1_ but increased RV and RV/TLC^10^, therefore, the central phenomenon of this pattern is the increase in RV and RV/TLC, which resulted in the concomitant decrease of VC and FEV_1_ and the lack of correlation between FEV_1_ and asthma severity due to the following potential mechanisms: 1) loss of elastic recoil (extrinsic obstruction), airways closed at the same transpulmonary pressure as in healthy people but at a higher lung volume, which limited further emptying of the lungs^25^; 2) small airways obliteration (intrinsic obstruction), small airways narrowed at a higher transpulmonary pressure that in healthy people, which limited further expiration^26^.

Meanwhile, studies have also demonstrated that in many patients with asthma, lung volume is increased. Sorkness and his colleagues analyzed the plethysmographic lung function in patients with no asthma, nonsevere asthma, and severe asthma; and they found that RV, TLC, and ratio of RV to TLC (RV/TLC) were significantly higher in patients with severe asthma than in patients without asthma, and that air trapping was a characteristic feature of severe asthma population^27^. Furthermore, Hartley *et al*. have elucidated that air trapping was significantly increased in patients with asthma, and multiple regression analyses showed that in asthma subgroups with postbronchodilator predicted percentage of forced expiratory volume (FEV_1_%pred) less than 80%, air trapping was a strong predictor of lung function impairment^28^. In addition, a recent study found that higher RV/TLC was a significant determinant of decreased deep inspiration-induced bronchodilation, which was strongly correlated with lower FEV_1_ and airway distensibility^29^. In the present study, we also found that lung volumetric parameters (RV, TLC and RV/TLC) varied significantly in asthmatics with different severity levels and revealed a significant negative correlation between lung volumes and FEV_1_%pred. Hence, it seems reasonable to believe that lung volumetric parameters have potential diagnostic values in distinguishing asthmatic severity.

For the first time, to our knowledge, our study specifically evaluated the roles of lung volumetric parameters in differentiating levels of asthma severity. In an attempt to determine whether lung function measures were consistent with levels of asthma severity, Bacharier and his colleagues classified asthma severity via symptom frequency and medication use in 219 asthmatic children^7^. Although FEV_1_%pred did not differ by severity, as we cited above, they found a significant negative correlation between asthma severity and ratio of FEV_1_ to forced vital capacity (FEV_1_/FVC) (mild: 86.3 ± 8.5 vs. moderate: 83.0 ± 10.3 vs. severe: 79.8 ± 11.8, P < 0.001). They attributed such a negative correlation to the dysanapsis, an incongruence between the growth of the airways and lung parenchyma, which worsened in asthma with airways smaller than the lung parenchyma and could resulted in higher lung volumes; but they did not examine related parameters such as RV or RV/TLC. In our study, we found that RV%pred measured by BP and ΔTLC%pred, with a high sensitivity ranging from 77.78% to 97.22% and specificity between 52.94% and 73.53%, could effectively differentiate asthma severity. We also detected that TLC% measured by BP had a sensitivity as high as 82.35% in distinguishing mild and moderate asthma. In discriminating moderate and severe asthma, we found a relatively low sensitivity of 63.89% with the *P* value being on the borderline, which might be attributed to the limited patient samples. By contrast, RV/TLC measured by BP, ΔRV%pred and ΔRV/TLC rendered high diagnostic values only in discriminating moderate and severe asthma, and the diagnostic specificity significantly surpassed the sensitivity (RV/TLC: 85.29% and 69.44%; ΔRV%pred: 85.29% and 58.33%), except for an equivalence in ΔRV/TLC (64.71% and 69.44%), which may result from the higher increase of gas trapping in severe asthma than that in mild asthma, as well as a similar increase of RV and TLC in mild asthma but a higher increase of RV than TLC in severe asthma. Nevertheless, a definitive conclusion of the influencing factors could not be drawn because we were unable to conduct spirometry in these patients before the onset of asthma to collect the baseline lung volumes.

Despite the findings, the present study had three limitations, which may need cautious interpretation of our results. For one thing, the number of participants is small, especially those with mild asthma, which may result in an inaccuracy of our findings. Secondly, the severity levels of asthma used in this study are based on outdated guidelines (NAEPP 1997), which lack assessment of beta2-agonist reliever use. The severity classification scheme better reflects what is currently termed asthma control according to the most recent guidelines introduced by the Global Initiative for Asthma (GINA). Thirdly, in addition to a diagnosis of asthma, a large proportion of the patients in this study have significant smoking history across all severity groups and persistent airflow limitation could not be entirely excluded, such that chronic obstructive pulmonary disease (COPD) may be a confounder. Finally, we used FEV_1_ to identify asthma severity in our study, which might not entirely uncover the true relationship among FEV_1_, gas trapping and asthma severity.

## Conclusions

In the diagnosis of asthma, body plethysmography is the optimal assessing method, and lung volumes assessed by body plethysmography as well as the difference between body plethysmography and helium dilution can differentiate between mild vs. severe, and moderate vs. severe asthma, but not mild vs. moderate asthma. Nevertheless, future investigations are still warranted to further reveal the correlation of lung volumes and asthma functional outcomes such as asthma controls, and to confirm the clinical applicability of lung volumes in discriminating asthma severity in clinical settings.

## Methods

Study protocol was approved by the Institutional Ethical Committee for Clinical and Biomedical Research of West China Hospital (Sichuan, China), and all participants provided written informed consent. All methods were performed in accordance with the relevant guidelines and regulations released by the Chinese National Institutes of Health and the Clinical Trial Center of West China Hospital.

### Patients

From January 2014 to April 2015, consecutive outpatients with chronic persistent asthma were enrolled in West China Hospital, Sichuan University. The diagnostic criteria of asthma were recommended by the Global Initiative for Asthma (GINA) 2012 guideline including: 1) recurrent respiratory symptoms with episodic breathlessness, wheeze, cough, and chest tightness, which were often triggered by incidental allergen exposure, seasonal variability and upper respiratory tract infection, and might resolve spontaneously or in response to medication; 2) airflow reversibility or airway responsiveness documented by lung function test, which was an increase in FEV_1_ of >12% and >200 ml from baseline or a diurnal variation if peak expiratory flow (PEF) of >20% or a fall in FEV_1_ from baseline of ≥20% with standard doses of methacholine^22^. We excluded patients with additional obstructive lung diseases, such as COPD, asthma-COPD overlap syndrome (ACOS), and cystic fibrosis.

Chronic persistence was defined as onset of asthmatic symptoms every week with various frequency or extent, and three severity levels were classified in accordance with the guideline released by National Asthma Education and Prevention Program (NAEPP) on the basis of daytime symptoms, night waking, activity limitation due to asthma, and FEV_1_%pred^21^: mild persistent (daytime symptoms ≥1 episode/week but not daily presenting, night waking >2 episode/month but <1 episode/week, probable activity limitation, and FEV_1_%pred >80%), moderate persistent (daily daytime symptoms, night waking ≥1 episode/week, activity limitation, and 60% ≤ FEV_1_%pred ≤ 80%) and severe persistent (daily daytime symptoms, frequent night waking, activity limitation, and FEV_1_%pred <60%).

### Lung function test

To detect lung volumes (RV, TLC), BP and HD were performed in all enrolled patients using a calibrated equipment (JAEGER, Jaeger Corp, Germany), consisting of a mixing fan, carbon dioxide (CO_2_) absorber, oxygen (O_2_) and helium supply, a gas inlet and outlet, and a water vapor absorber, and followed by MasterScreen Pulmonary Function Test (PFT) System to measure spirometry (FEV_1_, PEF).

All test procedures complied with the standardizations recommended by American Thoracic Society/European Respiratory Society (ATS/ERS) guidelines^12^,^30^. BP contained a series of gentle pants at a frequency between 0.5 and 1.0 Hz to calculate lung volumes. When HD was used, patients were instructed to breathe for 30–60 seconds, and then switched them to the helium gas (turn in). The helium concentration was recorded every 15 seconds until the helium equilibration was complete (i.e. change of helium concentration is <0.02% for 30 seconds). The patients were finally disconnected from the helium gas (turn out). Spirometry was conducted via three distinct phases to depict the flow-volume curves including: 1) maximal inspiration; 2) a “blast” of exhalation; and 3) continued complete exhalation until the volume-time curve showed no change in volume (<0.025 L) for ≥1 s and the subject had tried to exhale for ≥6 s.

Spirometrics were detected as predicted percentage of FEV_1_ (FEV_1_%pred), PEF (PEF%pred), and maximal mid-expiratory flow (MMEF%pred); while lung volumes were displayed as predicted percentage of RV (RV%pred) and TLC (TLC%pred), and RV/TLC. Differences in lung volumes between BP and HD were calculated as predicted percentage of RV (ΔRV%pred), TLC (ΔTLC%pred) and RV/TLC (ΔRV/TLC).

### Statistical analysis

Continuous data were reported as mean and standard deviation (SD), while dichotomous data were reported as frequency and proportion. SPSS 21.0 (Copyright (c) SPSS Inc. 1989–2007) was used to test the hypothesis with a two-sided *P* value of <0.05 indicating statistical significance.

Baseline lung function measures, such as FEV_1_%pred, PEF%pred, MMEF%pred, RV%pred, TLC%pred, RV/TLC, ΔRV%pred, ΔTLC%pred, and ΔRV/TLC difference, were compared among three severity levels using *One-way Analysis of Variance (ANOVA)* and *Least-Significant Difference (LSD) posthoc tests. Student-t test* was conducted to compare the RV%pred, TLC%pred and RV/TLC between BP and HD. Correlation analysis was performed to calculate *r* value between lung volumetric parameters (RV%pred, TLC%pred, RV/TLC, ΔRV%pred, ΔTLC%pred, and ΔRV/TLC) and FEV_1_%pred. We depicted receiver operating characteristic (ROC) curves and calculated area under the curve (AUC) to evaluate the accuracy of RV%pred, TLC%pred, RV/TLC, ΔRV%pred, ΔTLC%pred, and ΔRV/TLC in discriminating different asthmatic severity levels. Cutoff points were defined as the point when Youden’s index (=sensitivity + specificity-1) reached the maximum, and the sensitivity, specificity, positive predictive values (PPVs), negative predictive values (NPVs) as well as likelihood ratios (LRs) were also calculated in different severity levels.

## Additional Information

**How to cite this article:** Luo, J. *et al*. Clinical Roles of Lung Volumes Detected by Body Plethysmography and Helium Dilution in Asthmatic Patients: A Correlation and Diagnosis Analysis. *Sci. Rep.* **7**, 40870; doi: 10.1038/srep40870 (2017).

**Publisher's note:** Springer Nature remains neutral with regard to jurisdictional claims in published maps and institutional affiliations.

## Figures and Tables

**Table 1:** Baseline characteristics in patients with mild, moderate and severe asthma.

Parameters	Mild (n = 23)	Moderate (n = 34)	Severe (n = 36)	*P* (ANOVA)	*P* (Mild vs. Moderate)	*P* (Mild vs. Severe)	*P* (Moderate vs. Severe)
**Demographics**							
Age (years)	52.0 ± 13.5	53.4 ± 12.6	54.4 ± 12.4	0.777	0.676	0.479	0.749
Gender (Male, %)	6 (26.1)	18 (52.9)	24 (66.7)	0.010	0.058	0.003	0.330
Duration of asthma (year)	4.5 ± 4.0	4.7 ± 2.5	6.1 ± 2.9	0.083	0.820	0.056	0.060
Smoking history (pack*year)	6.7 ± 8.7	10.5 ± 12.4	10.9 ± 10.8	0.321	0.207	0.157	0.874
**Spirometric parameters**							
Pre-bronchodilator FEV_1_%pred (%)	84.1 ± 3.3	70.6 ± 4.4	51.4 ± 6.6	<0.001	<0.001	<0.001	<0.001
FEV_1_/FVC (%)	64.6 ± 10.2	59.6 ± 8.2	50.5 ± 9.2	<0.001	0.044	<0.001	<0.001
% Change of FEV_1_ (%) (in airway responsibility test)	29.74 ± 10.52	28.67 ± 13.76	27.88 ± 13.51	0.865	0.759	0.592	0.800
FEV_1_ Change (mL) (in airway responsibility test)	357.39 ± 118.02	384.71 ± 171.29	334.72 ± 131.44	0.355	0.486	0.558	0.152
PEF%pred (%)	70.1 ± 16.9	68.4 ± 11.1	50.0 ± 11.7	<0.001	0.633	<0.001	<0.001
MMEF%pred (%)	33.7 ± 12.3	26.9 ± 9.9	17.4 ± 7.3	<0.001	0.011	<0.001	<0.001
**Volumetric parameters**							
RVpleth%pred (%)	139.0 ± 38.1	164.2 ± 24.5	198.1 ± 37.8	<0.001	0.007	<0.001	<0.001
TLCpleth%pred (%)	108.8 ± 15.6	120.2 ± 12.2	127.0 ± 13.3	<0.001	0.002	<0.001	0.040
RV/TLCpleth (%)	45.9 ± 7.5	47.2 ± 7.2	56.4 ± 9.1	<0.001	0.550	<0.001	<0.001
RVhe%pred (%)	87.8 ± 18.6	100.0 ± 20.0	108.5 ± 28.6	0.006	0.057	0.001	0.133
TLChe%pred (%)	92.7 ± 13.8	97.5 ± 11.3	94.5 ± 13.5	0.359	0.170	0.609	0.326
RV/TLChe (%)	37.2 ± 8.0	36.4 ± 7.8	40.6 ± 8.4	0.079	0.724	0.116	0.033
ΔRV%pred (%)	51.1 ± 34.8	64.2 ± 24.3	89.6 ± 39.3	<0.001	0.150	<0.001	0.002
ΔTLC%pred (%)	15.2 ± 7.0	23.3 ± 9.5	34.2 ± 9.8	<0.001	0.001	<0.001	<0.001
ΔRV/TLC (%)	8.7 ± 8.6	10.8 ± 6.4	15.8 ± 8.2	0.002	0.319	0.001	0.008

ANOVA, analysis of variance; FEV_1_, forced expiratory volume in one second; FEV_1_%pred, predicted percentage of forced expiratory volume in one second; FEV_1_/FVC, ratio of forced expiratory volume in one second to forced vital capacity; MMEF%pred, predicted percentage of maximal mid-expiratory flow; PEF%pred, predicted percentage of peak expiratory flow; RVhe%pred, predicted percentage of residual volume measured by helium dilution method; RVpleth%pred, predicted percentage of residual volume measured by body plethysmography; RV/TLChe, ratio of residual volume to total lung capacity measured by helium dilution method; RV/TLCpleth, ratio of residual volume to total lung capacity measured by body plethysmography; TLChe%pred, predicted percentage of total lung capacity measured by helium dilution method; TLCpleth%pred, predicted percentage of total lung capacity measured by body plethysmography; ΔRV%pred, predicted percentage of difference of residual volume between body plethysmography and helium dilution method; ΔRV/TLC, difference of ration of residual volume to total lung capacity between body plethysmography and helium dilution method; ΔTLC%pred, predicted percentage of difference of total lung capacity between body plethysmography and helium dilution method.

**Table 2:** Correlation analysis between lung volumes and FEV_1_%pred.

Lung volumetric parameters	*r*	*P*
RVpleth%pred	−0.557	<0.001
TLCpleth%pre	−0.387	<0.001
RV/TLCpleth	−0.485	<0.001
RVhe%pred	−0.245	0.018
TLChe%pre	0.089	0.396
RV/TLChe	−0.176	0.091
ΔRV%pred	−0.457	<0.001
ΔTLC%pre	−0.668	<0.001
ΔRV/TLC	−0.375	<0.001

FEV_1_%pred, predicted percentage of forced expiratory volume in one second; RVhe%pred, predicted percentage of residual volume measured by helium dilution method; RVpleth%pred, predicted percentage of residual volume measured by body plethysmography; RV/TLChe, ratio of RV to TLC measured by helium dilution method; RV/TLCpleth, ratio of RV to TLC measured by body plethysmography; TLChe%pred, predicted percentage of total lung capacity measured by helium dilution method; TLCpleth%pred, predicted percentage of total lung capacity measured by body plethysmography; ΔRV%pred, predicted percentage of difference of residual volume between body plethysmography and helium dilution method; ΔRV/TLC, difference of ration of residual volume to total lung capacity between body plethysmography and helium dilution method; ΔTLC%pred, predicted percentage of difference of total lung capacity between body plethysmography and helium dilution method.

**Table 3:** Cut-off points of RVpleth%pred and ΔRV%pred, TLCpleth%pred and ΔTLC%pred, RV/TLCpleth and ΔRV/TLC, and the corresponding sensitivity, specificity, PPV, NPV and LR in distinguishing different asthma severity levels.

	Cut-off point	Sensitivity	Specificity	PPV	NPV	LR+	LR−
**Mild vs. Moderate**							
RVpleth%pred	145.4	79.41	65.22	77.14	68.18	2.28	0.32
TLCpleth%pred	108.7	82.35	47.83	70.00	64.71	1.58	0.37
RV/TLCpleth	45.8	61.76	56.52	67.74	50.00	1.42	0.68
ΔRV%pred	42.0	85.29	43.48	69.05	66.67	1.51	0.34
ΔTLC%pred	14.2	88.24	56.52	75.00	76.47	2.03	0.21
ΔRV/TLC	3.1	91.18	30.43	65.96	70.00	1.31	0.29
**Moderate vs. Severe**							
RVpleth%pred	179.9	77.78	73.53	75.68	75.76	2.94	0.30
TLCpleth%pred	123.4	63.89	67.65	67.65	63.89	1.97	0.53
RV/TLCpleth	53.1	69.44	85.29	83.33	72.50	4.72	0.36
ΔRV%pred	88.5	58.33	85.29	80.77	65.91	3.97	0.49
ΔTLC%pred	20.2	97.22	52.94	68.63	94.74	2.07	0.05
ΔRV/TLC	11.7	69.44	64.71	67.57	66.67	1.97	0.47

LR, likelihood ratio; NPV, negative predictive value; PPV, positive predictive value; RVpleth%pred, predicted percentage of residual volume measured by body plethysmography; RV/TLCpleth, ratio of RV to TLC measured by body plethysmography; TLCpleth%pred, predicted percentage of total lung capacity measured by body plethysmography; ΔRV%pred, predicted percentage of difference of residual volume between body plethysmography and helium dilution method; ΔRV/TLC, difference of ration of residual volume to total lung capacity between body plethysmography and helium dilution method; ΔTLC%pred, predicted percentage of difference of total lung capacity between body plethysmography and helium dilution method.
